# Desulfonylative Functionalization of Organosulfones via Inert (Hetero)Aryl C(*sp^2^*)–SO_2_ Bond Cleavage

**DOI:** 10.3390/molecules29174137

**Published:** 2024-08-31

**Authors:** Rui Huang, Boning Gu, Ming Wang, Yinsong Zhao, Xuefeng Jiang

**Affiliations:** 1 State Key Laboratory of Estuarine and Coastal Research, Hainan Institute of East China Normal University, East China Normal University, Shanghai 200241, China; 2 State Key Laboratory of Petroleum Molecular & Process Engineering, Shanghai Key Laboratory of Green Chemistry and Chemical Processes, School of Chemistry and Molecular Engineering, East China Normal University, Shanghai 200062, China

**Keywords:** (hetero)arylsulfones, desulfonylative functionalization, C(*sp^2^*)–SO_2_ bond cleavage, transition-metal catalysis, photocatalysis

## Abstract

As “chemical chameleons,” organosulfones have been widely applied in various desulfonylative functionalization reactions. However, the desulfonylative functionalization of (hetero)arylsulfones through the cleavage of inert C(*sp^2^*)–SO_2_ bonds remains a challenging and underexplored task. Over the past twenty years, the use of (hetero)arylsulfones as arylation reagents has gradually gained attention in diverse cross-coupling reactions under specific catalytic conditions, especially in transition metal-catalysis and photocatalysis chemistry. In this review, we discuss the representative accomplishments and mechanistic insights achieved in desulfonylative reactions of inactive C(*sp^2^*)–SO_2_ bonds in (hetero)arylsulfones, including: (i) transition-metal-catalyzed desulfonylative cross-coupling reactions and (ii) photo-/electrocatalytic radical desulfonylative coupling reactions. We anticipate that this review will provide an overall perspective in this area to a general audience of researchers and stimulate further innovative strategies for desulfonylative functionalization of inert arylsulfones.

## 1. Introduction

Organosulfones are highly versatile building blocks widely utilized in pharmaceutical science, functional materials, and polymer science [1,2,3]. Known as “chemical chameleons”, organosulfones have participated in a diverse array of reactions, enabling the valuable formations of new chemical bonds [1,4]. The desulfonylative functionalization of organosulfones—substituting the sulfonyl group with other functional groups—is an attractive alternative that enhances molecular complexity [5]. Owing to its strong electron-withdrawing ability and inert reactivity, the sulfonyl functionality typically serves as a leaving group in elimination transformations or as an auxiliary moiety to stabilize carbanions in traditional organic synthesis. Over the past decades, studies on organosulfones have garnered significant attention, focusing on utilization as organic (pseudo)halides in cross-coupling reactions [6,7,8,9,10,11]. By integrating novel catalytic methods and customizing substituents on organosulfones, numerous new methodologies have emerged to activate C–SO_2_ bonds, leading to remarkable advancements in desulfonylative functionalizations, even in innovative transformations that are challenging to achieve with conventional electrophiles or nucleophiles. Despite several excellent reviews on C–S bond functionalization [9] and radical desulfonylative functionalization [6,7,10,11] through transition-metal catalysis or photocatalysis, which highlight the cleavage of aryl, alkyl, vinylic, and alkynyl C–SO₂ bonds in thioethers, sulfamides, sulfonates, sulfones, and their derivatives, there is a limited number of reviews that focus specifically on the desulfonylative functionalization of organosulfones via inert (hetero)aryl C(*sp^2^*)–SO₂ bond cleavage [8].

**Figure 1:**
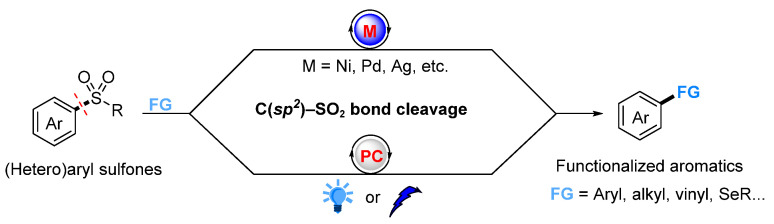
Desulfonylative functionalization of (hetero)arylsulfones via C(*sp^2^*)–SO_2_ bond cleavage.

In this review, we will succinctly recount the history of desulfonylative functionalization of inactive (hetero)arylsulfones, focusing on representative accomplishments and progress as arylation reagents to access functionalized aromatics. The review is divided into two main sections: (i) transition-metal-catalyzed desulfonylative cross-coupling reactions and (ii) photo-/electrocatalytic desulfonylative coupling reactions (Figure 1).

## 2. Transition Metal-Catalyzed Desulfonylative Cross-Coupling Reaction

### 2.1. Kumada-Type Cross-Coupling Reaction

**Scheme 1:**
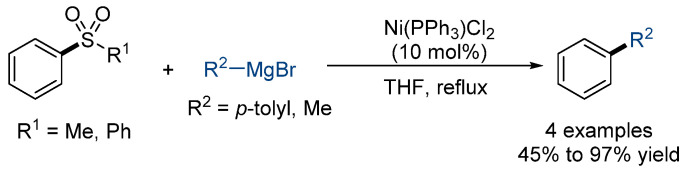
Seminal desulfonylative cross-coupling of arylsulfones with Grignard reagents.

In 1979, Wenkert and co-workers reported the first Ni-catalyzed Kumada-type cross-coupling reaction of phenyl sulfones with methyl/*p*-tolyl magnesium bromides, giving desulfonylative methylation/*p*-tolylation products in medium to high yields (Scheme 1) [12]. When methyl phenyl sulfone was tested under the optimized conditions, the insertion of Ni(0) complex into the aryl C(*sp^2^*)–SO_2_ bond rather than the methyl C(*sp^3^*)–SO_2_ bond occurred selectively. This seminal achievement indicated the possibility of substituted arylsulfones as (pseudo)halide reagents in cross-coupling reactions.

**Scheme 2:**
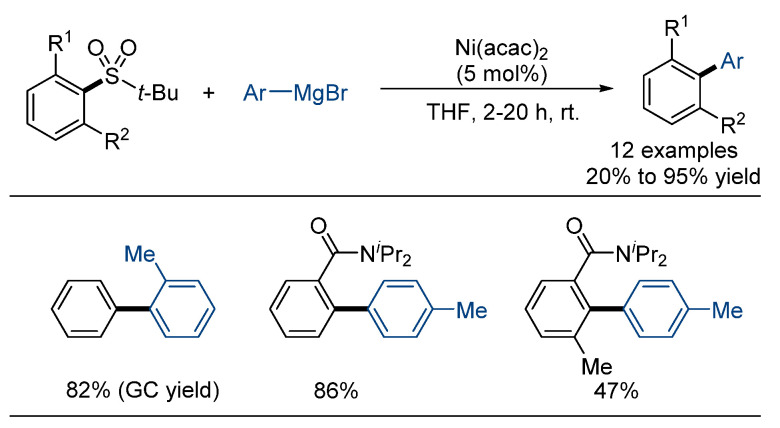
Ni-catalyzed cross-coupling of aryl sulfones with Ar-MgBr.

In the 1990s, Clayden and Julia developed a modified Ni-catalyzed cross-coupling reaction of aryl *tert*-butylsulfones with aryl magnesium halides (Scheme 2) [13,14]. This protocol established expanded scopes of arylsulfones and Grignard reagents, yielding various *ortho*-substituted unsymmetrical biaryls in satisfactory yields. However, mixtures of cross-coupling, homocoupling, and reduction products were also afforded due to the steric effect of *ortho*-substituents.

**Scheme 3:**
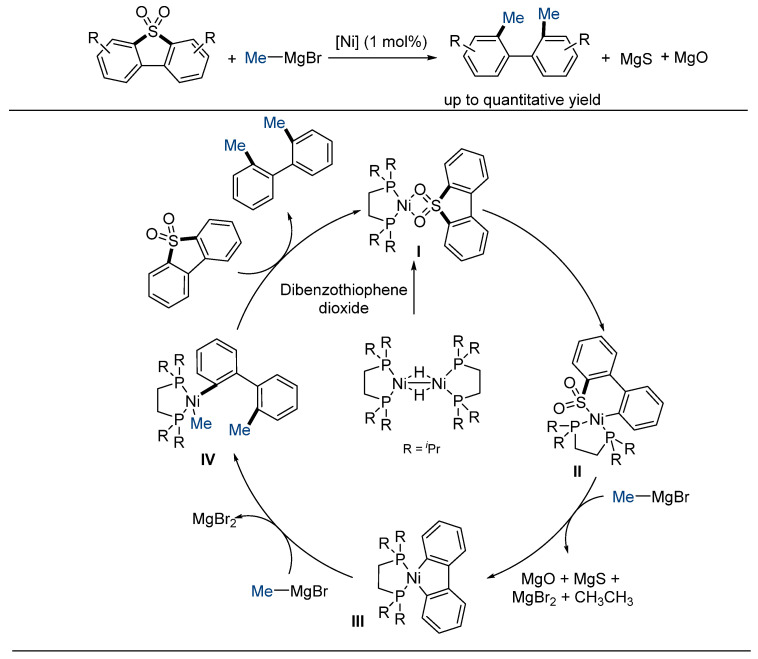
Ni-catalyzed desulfonylative cross-coupling of dibenzothiophene dioxides.

In 2008, García and co-workers developed the desulfonylative functionalization of dibenzothiophene dioxides with methyl magnesium bromide (MeMgBr) catalyzed by nickel-complexes (Scheme 3) [15]. A series of nickel complexes including [Ni(dippe)H]_2_, [Ni(dcype)H]_2_, [Ni(dtbpe)H]_2_, or [Ni(dippe)(Me)_2_] in mixed solvents of toluene and THF could yield methyl biaryl or dimethyl biaryls in moderate to good yields. Dibenzothiophene dioxides reacted with 6.0 equivalents of MeMgBr to give dimethyl biaryls in quantitative yields in the presence of low nickel catalyst loading (1.0 mol%). As shown in the proposed catalytic cycle, the reaction of [Ni(dippe)H]_2_ with dibenzothiophene dioxide yields a nickel-complex **I**. Further oxidative addition of C(*sp^2^*)–SO_2_ bond followed by extrusion of SO_2_ with the aid of MeMgBr furnishes cyclic nickel intermediate **III**. The intermediate **III** undergoes metathesis with MeMgBr followed by reductive elimination to give dimethyl-substituted biaryls.

**Scheme 4:**
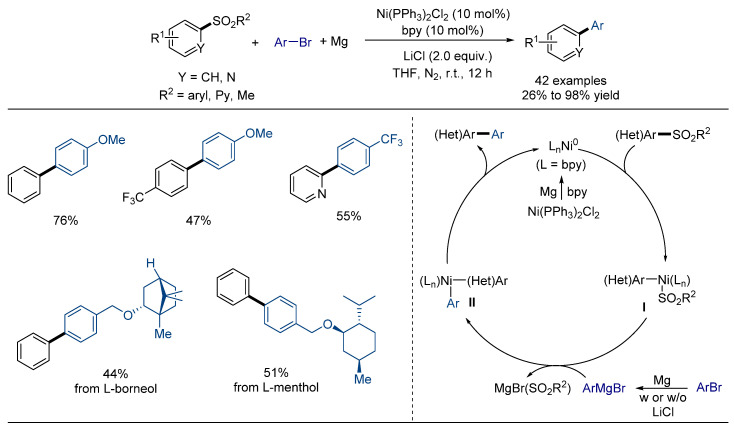
Ni-catalyzed desulfonylative cross-coupling with in situ-generated Ar-MgBr.

Very recently, Xu, Zhou, and Shen’s group established a Ni-catalyzed cross-coupling of inactivated (hetero)aryl sulfones with aryl bromides at ambient temperature with the assistance of excess magnesium powder (Scheme 4) [16]. Control experiments indicated that Ni(PPh_3_)_2_Cl_2_, 2,2′-bipyridine ligand (bpy), magnesium powder, and THF are all indispensable for the successful realization of the reaction. A variety of (hetero)aryl sulfones and aryl bromides were well compatible under the optimized conditions with/without LiCl, enabling the facile synthesis of biaryl compounds with moderate to high yields. It was noteworthy that the late-stage desulfonylative coupling reactions with complex molecules derived from *L*-borneol and *L*-menthol also proceeded smoothly under the established conditions, affording the corresponding product in moderate yields. In addition, the practical utility of this cross-coupling was demonstrated by performing a 5 mmol scale reaction, delivering the final products in 66% yield. For unsymmetrical (hetero)aryl sulfones, the desulfonylative cross-coupling reaction occurred selectively through the cleavage of the more electron-poor aryl C(*sp^2^*)–SO_2_ bond. Initially, the reduction of Ni(PPh_3_)_2_Cl_2_ by Mg powder generates active Ni(0) species, which undergo oxidative addition with aryl sulfones (ArSO_2_R^2^) to produce an Ar–Ni(II)–SO_2_R^2^ complex **I**. Meanwhile, the reaction of aryl bromides with Mg powder with/without the aid of lithium chloride produces the active aryl magnesium compounds. The subsequent transmetallation between intermediate **I** and Grignard reagents affords the biaryl-nickel intermediates **II**, which finally undergo reductive elimination to generate the desired diaryls.

### 2.2. Suzuki–Miyaura-Type Cross-Coupling Reaction

**Scheme 5:**
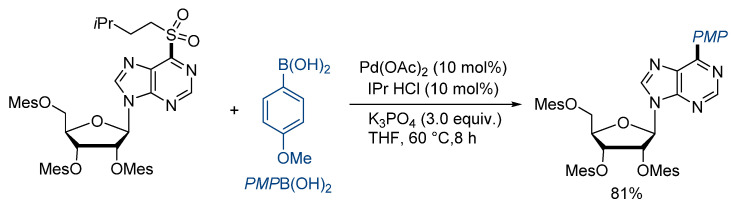
Pd-catalyzed cross-coupling of the sulfonyl-purine with *PMP*B(OH)_2_.

In 2005, the Robins group reported one example of Pd-catalyzed Suzuki–Miyaura-type cross-coupling of 6-[(3-methylbutyl)sulfonyl]-9-[2,3,5-tri-O-(2,4,6-trimethylbenzoyl)-β-D-ribofuranosyl]purine with 4-methoxyphenylboronic acid (*PMP*B(OH)_2_) [17]. In the presence of 10 mol% of Pd(OAc)2 and 10 mol% of 1,3-bis(2,6-diisopropylphenyl)imidazolin-2-ylidene (IPr), the corresponding desulfonylative arylation product was isolated in 81% yield. The cleavage of heteroaryl C(*sp^2^*)–SO_2_ bond preferred to occur (Scheme 5).

**Scheme 6:**
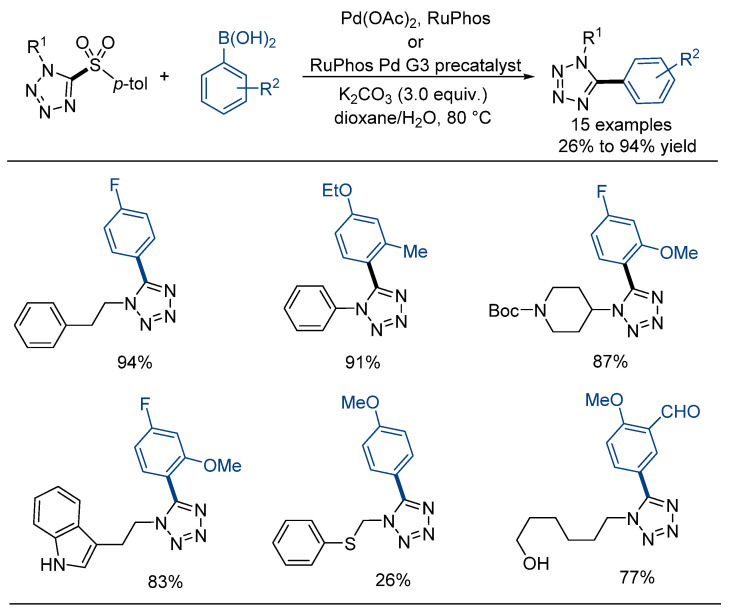
Pd-catalyzed cross-coupling of *N*-heteroarylsulfones with boronic acids.

Twelve years later, the possibility of *N*-heteroaryl sulfones to participate in Pd-catalyzed Suzuki–Miyaura-type cross-coupling reaction was further investigated by Hennessy and co-workers (Scheme 6) [18]. The synthesis of 1,5-disubstituted tetrazoles was successfully realized through desulfonylative cross-coupling of readily prepared 5-*p*-toluenesulfonyltetrazoles with boron-based nucleophiles including boronic acids, pinacolboronate esters, and trifluoroborate salts. The use of biaryl phosphine ligands such as RuPhos was essential for achieving higher yields. The protocol is compatible with a variety of 5-*p*-toluenesulfonyltetrazoles and organoboron compounds with diverse functional groups. It is noteworthy that a 5-*p*-tolylsulfonyltetrazole substrate containing the bromoaryl group underwent Pd-catalyzed Suzuki–Miyaura cross-coupling reaction selectively through the cleavage of aryl C–Br bond, thus providing new opportunities for step-wise construction of polyaryls.

**Scheme 7:**
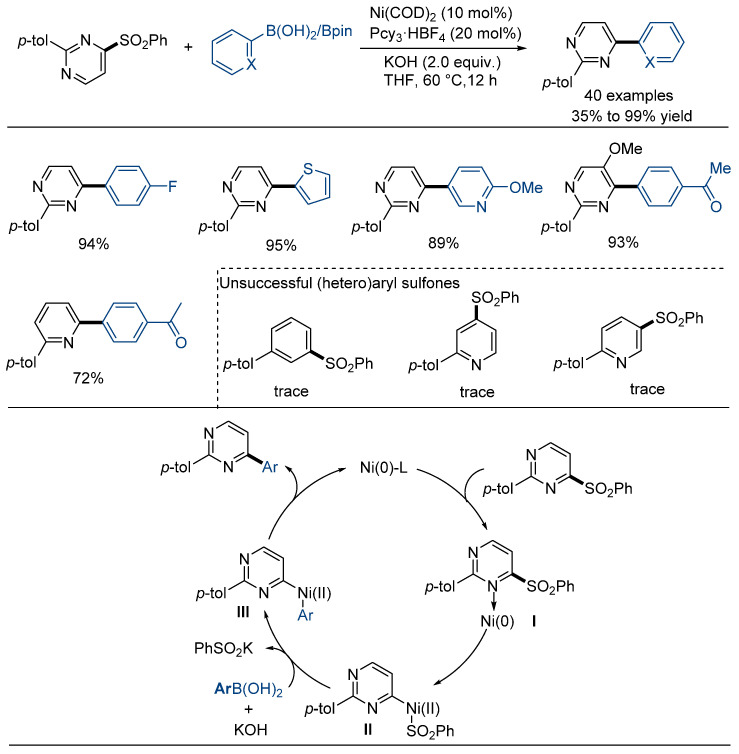
Ni-catalyzed cross-coupling of phenyl pyrimidinyl sulfones with boronic acids.

Very recently, Niu and co-workers reported a Ni-catalyzed Suzuki–Miyaura-type cross-coupling reaction of readily available and bench-stable phenyl pyrimidinyl sulfones with (hetero)arylboronic acids (Scheme 7) [19]. This desulfonylative cross-coupling reaction involving the pyrimidinyl C(*sp^2^*)–SO_2_ bond cleavage offered a facile route for the rapid synthesis of synthetically challenging 2,4-diarylated pyrimidines with moderate to high yields. The presence of an *ortho*-nitrogen atom in sulfone substrates is critical in this method. In the proposed catalytic cycle, the ligand exchange of Ni(0) species with phenyl pyrimidinyl sulfones first generates the intermediate **I**. Then, a chemoselective C(*sp^2^*)–SO_2_ bond insertion affords the oxidative additive Ni(II) complex **II**. Transmetallation with arylboronic acids furnishes the pyrimidinyl-Ni(II)-aryl complex **III**, which undergoes reductive elimination to give the final product.

**Scheme 8:**
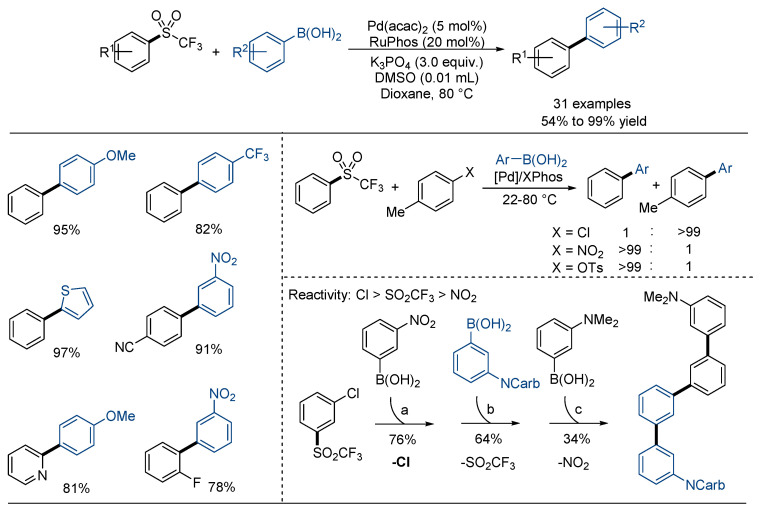
Pd-catalyzed cross-coupling of trifluoromethyl arylsulfones with boronic acids.

Compared to *N*-heteroaryl C(*sp^2^*)–SO_2_ bond, the activation of inert aryl C(*sp^2^*)–SO_2_ bond in arylsulfones is much more challenging due to its weak coordinating ability and higher bond dissociation energy. In 2019, the Moran group described the Suzuki–Miyaura cross-coupling using the trifluoromethyl arylsulfones as the substrates (Scheme 8) [20]. Control experiments indicated that the desulfonylative reactions of diphenyl sulfone, phenyl methyl sulfone and its mono- and difluorinated analogs were inert or much less reactive, suggesting the important role of the trifluoromethyl group in promoting aryl C(*sp^2^*)–SO_2_ bond activation. In addition, the presence of a small amount of DMSO (1%, v/v) was critical to obtain high yields by solubilizing the inorganic base. This protocol could provide various functionalized di(hetero)aryls from readily available trifluoromethyl arylsulfones and arylboronic acids. Furthermore, the reactivity of different electrophiles such as aryl chlorides, nitroarenes, and aryl tosylates was investigated in one pot. The reactivity of aryl sulfones showed at least two orders of magnitude slower than aryl chlorides, but at least two orders faster than nitroarenes and aryl tosylates under standard conditions, which provided new opportunities for multiple functionalization reactions in a step-wise manner. Mechanistic experiments and DFT calculations demonstrated that the relatively rare oxidative addition of palladium species into the aryl C(*sp^2^*)–SO_2_ bond was the turnover-limiting step.

In the same year, the Yorimitsu group developed similar desulfonylative transformations enabled by cooperative Pd/Rh catalysis [21]. Under the optimized condition, the model reaction could obtain 91% yield. In the absence of [Rh(cod)_2_]Cl_2_ catalyst, the reaction showed much poor efficiency (<40% yield) and higher loadings of Pd catalyst was required. Kinetic experiments indicated that the rhodium catalyst probably promoted the transfer of the aryl ring from arylboronate to palladium species, thus accelerating the transmetalation process on the palladium center, and the reductive elimination step to form C–C bond assisted by IPr^NiPr2^ ligand might be the turnover-limiting step. Despite the acyclic arylsulfones, the Suzuki–Miyaura type cross-coupling reaction of dibenzothiophene sulfones with arylboronic acids were achieved by García and co-workers. However, stoichiometric amounts of nickel salts were needed in this reaction, yielding complex desulfonylative arylation products [22].

### 2.3. Desulfonylative Cross-Coupling Reaction with (Hetero)Aryl Halides

**Scheme 9:**
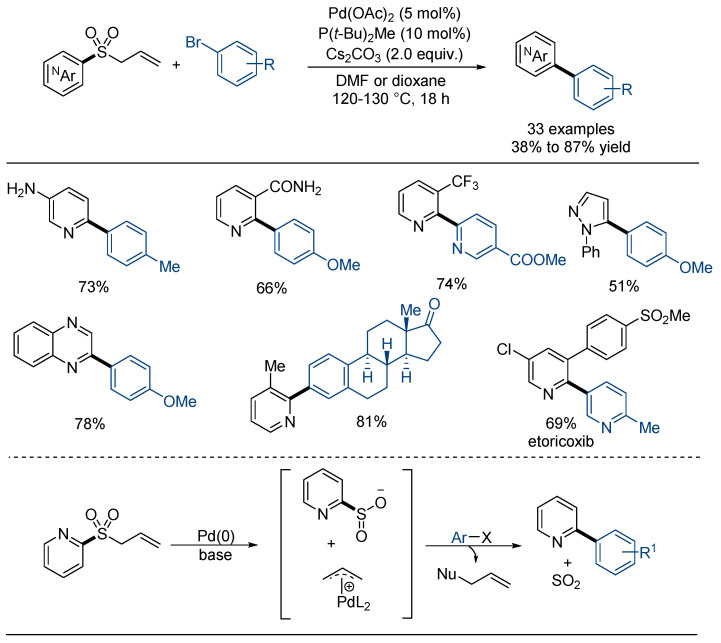
Pd-catalyzed cross-coupling of allyl heteroaryl sulfones with aryl halides.

As a class of organic (pseudo)halides, the desulfonylative cross-coupling of sulfones with electrophiles such as aryl halides has remained underexplored. In 2018, the Willis group demonstrated that *N*-heteroaryl allylsulfones could act as precursors of sulfinate reagents under palladium(0) catalysis, leading to efficient Pd-catalyzed cross-coupling reactions with (hetero)aryl halides in one pot (Scheme 9) [23]. Under the optimized conditions, the constructions of synthetically challenging *N*-heteroaryl-(hetero)aryl products were realized efficiently. Notably, the desulfonylative cross-coupling reaction showed high regioselectivity. In the proposed catalytic cycle, the oxidative addition reaction of active Pd(0) species with allylsulfones would generate the *π*-allyl-Pd(II) intermediate with the release of *N*-heteroaryl sulfinate. The π-allyl-Pd(II) intermediate was intercepted by a nucleophile to regenerate active Pd(0) species, thus allowing the subsequent cross-coupling of in situ-generated *N*-heteroaryl sulfinate with aryl halides to proceed. Later, the same group developed β-nitrile/ester alkyl (hetero)aryl sulfones as new latent sulfinate reagents [24]. In the presence of base, β-nitrile/ester alkyl (hetero)aryl sulfones could release the corresponding sulfinates and vinylic nitrile/ester by-product. These latent sulfinate reagents are stable and readily available, facilitating multistep elaborations that are challenging for traditional sulfinates with poor solubility and reactivity.

**Scheme 10:**
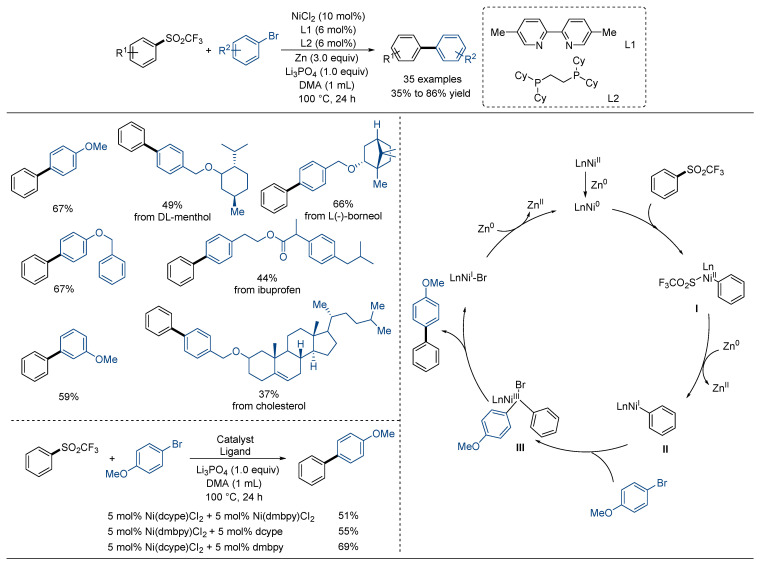
Ni-catalyzed desulfonylative cross-coupling of arylsulfones with aryl halides.

Very recently, the Ni-catalyzed reductive cross-coupling of arylsulfones with aryl bromides was realized by Ma and co-workers (Scheme 10) [25]. In this protocol, trifluoromethyl arylsulfones showed the best result, while other arylsulfones containing CF_2_H, CH_3_, Ph, and F instead of the CF_3_ group resulted in poor or no efficiency. By using 10 mol% of NiCl_2_ as the catalyst, the combination of 6 mol% of dmbpy and 6 mol% of dcype as ligands, and Zn powder as reductant, a variety of functionalized biaryl compounds were obtained with moderate to good isolated yields. However, aryl (trifluoromethyl) sulfones containing strong electron-withdrawing groups such as ketone, ester, and cyano groups failed to afford the desired product. In addition, the applicability of this method was then investigated using several aryl bromides derived from natural products and drug molecules such as ibuprofen and cholesterol, affording the desired products in moderate yields.

To gain insight into the mechanism, a series of control experiments were then conducted. Under the optimized conditions, the cross-coupling of phenyl (trifluoromethyl) sulfone with preformed phenylzinc bromide did not occur without Zn powder, excluding the possible formation of organozinc reagents in situ. The reaction of Ni-complex PMP-(dmbpy)Ni(II)-Br with arylsulfones gave no desired product, while the reaction of Ni-complex Ph-(dcype)Ni(II)–SO_2_CF_3_, generated from oxidative addition of aryl sulfone to Ni(0) species and confirmed by an X-ray analysis, could give the final product in 49% GC yield. However, the reaction of Ni-complex PMP-(dcype)Ni(II)-Br with arylsulfones also gave the desired product in 20% GC yield, thus the oxidative addition of aryl halides to nickel species could not be excluded. A possible catalytic cycle was also proposed. The key intermediate **II**, generated from intermediate **I** in the presence of Zn, inserts C–Br bond of aryl halides to give intermediate **III**. Reductive elimination of **III** gives the desired product.

### 2.4. Intramolecular Desulfonylative Cross-Coupling Reaction

**Scheme 11:**
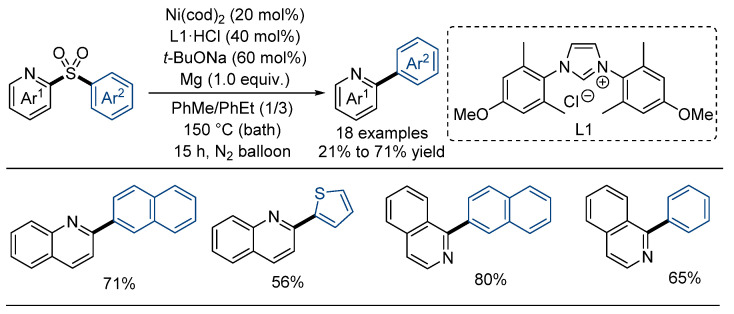
Ni-catalyzed intramolecular desulfonylative cross-coupling of *N*-hetero-2-aryl arylsulfones.

Without the addition of coupling reagents, transition metal-catalyzed intramolecular desulfonylative coupling reactions occurred in some cases. In 2018, Yorimitsu and co-workers developed a Ni-catalyzed intramolecular desulfonylative coupling of π-extended *N*-hetero-2-aryl arylsulfones (Scheme 11) [26]. In the presence of 20 mol% of Ni(cod)_2_, 40 mol% of ligand, 60 mol% of *t*-BuONa, and one equivalent of Mg, sulfones with π-extended *N*-aromatic rings and arylsulfonyl group at *ortho*-nitrogen carbon atom were well compatible to the corresponding unsymmetric biaryls with moderate to high yield. However, sulfones containing 2-pyridyl, 2-pyrazinyl, and 3-/4-quinolyl rings showed much lower efficiency or were completely inert under the optimized conditions.

**Scheme 12:**
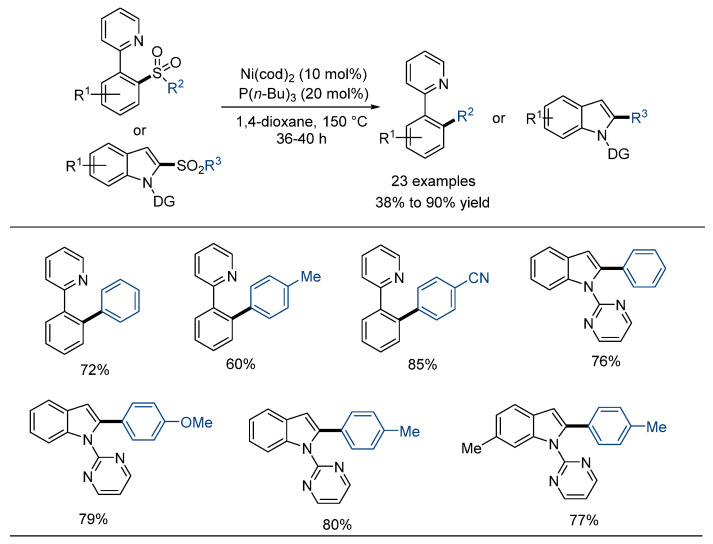
Ni-catalyzed intramolecular desulfonylative cross-coupling of *N*-hetero-2-aryl arylsulfones.

In 2019, the Wei group found that (hetero)aryl sulfones bearing an auxiliary group could undergo an intramolecular desulfonylative coupling reaction under nickel catalysis (Scheme 12) [27]. The strong coordinating 2-pyridyl and 2-pyrimidyl groups showed the best results. Using Ni(cod)_2_ as catalyst and P(*n*-Bu)_3_ as the ligand, a broad range of (hetero)biaryls were obtained with moderate to high yields. It was notable that the benzyl (hetero)aryl sulfone successfully gave the desired product in 74% yield through the cleavages of both C(*sp^2^*)–SO_2_ and C(*sp^2^*)–SO_2_ bonds. Without a directing group, the intramolecular desulfonylative coupling reaction also occurred, but required a stoichiometric amount of Ni(cod)_2_.

### 2.5. Desulfonylative Cross-Coupling Reaction with Other Reagents

**Scheme 13:**
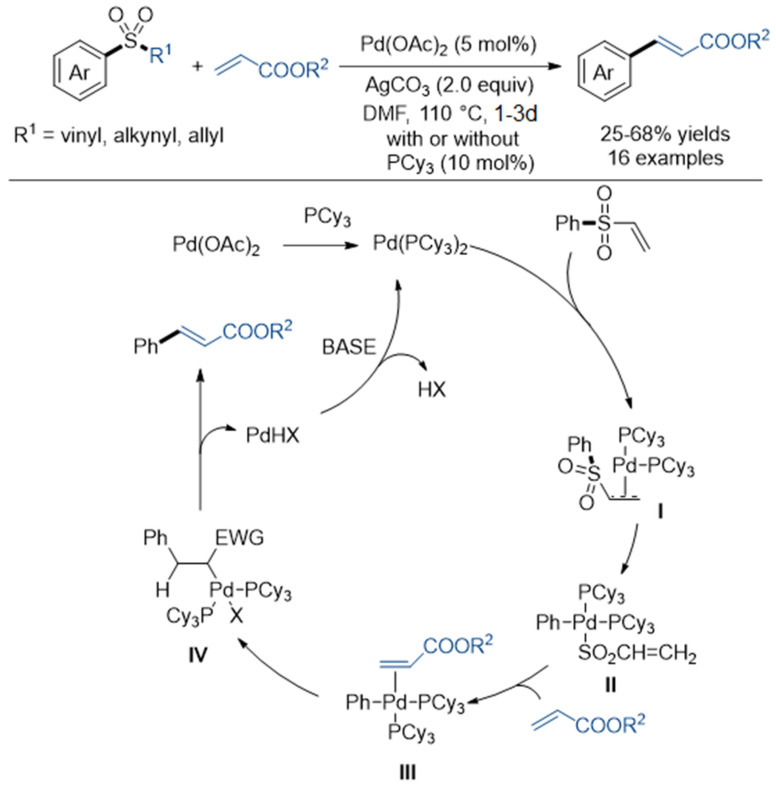
Pd-catalyzed Heck-type desulfonylative cross-coupling.

In 2006, Ruano and co-workers reported a Pd-catalyzed Mizoroki–Heck-type reaction of alkenyl or alkynyl aryl sulfones with acrylate esters (Scheme 13) [28]. By using Pd(OAc)_2_/PCy_3_ as the catalyst and Ag_2_CO_3_ as an additive, the cleavage of aryl C(*sp^2^*)–SO_2_ bond within alkenyl or alkynyl aryl sulfones proceeded primarily, leading to further arylation of acrylate esters to form cinnamyl esters. Diphenyl sulfone or methyl phenyl sulfone were unreactive under the optimal conditions, suggesting that coordination of the π-bond of the alkene and alkyne moiety to palladium center is likely involved. As shown in the proposed catalytic cycle, the coordination of the π-bonds followed by oxidative addition to Pd-species affords the intermediate **II**. Then, coordination and insertion of π-bond in the acrylate ester generates an alkyl-Pd species **III**, which undergoes the β-elimination process to give the desired product.

**Scheme 14:**
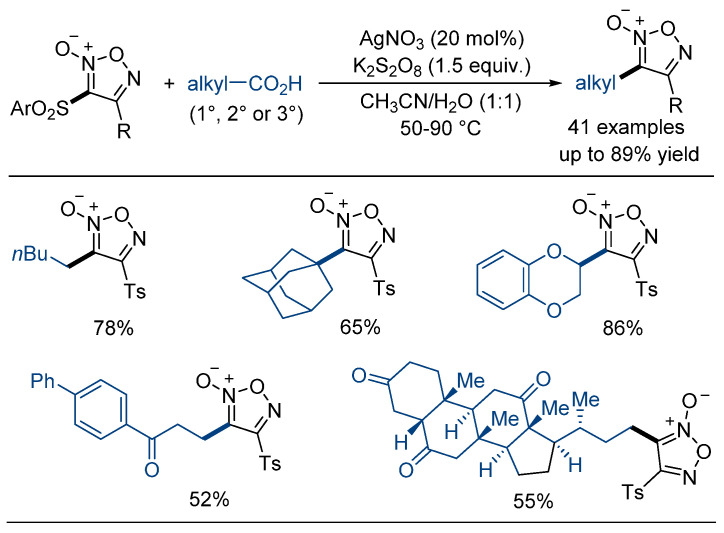
Ag-catalyzed desulfonylative cross-coupling with alkyl carboxylic acids.

Transition metal-catalyzed desulfonylative cross-coupling reactions of (hetero)aryl sulfones with radical precursors have also been achieved. In 2020, Matsubara and co-workers developed the Ag-catalyzed radical cross-coupling of arylsulfonyl-substituted furoxans with aliphatic carboxylic acids (Scheme 14) [29]. A variety of primary, secondary, and tertiary alkyl carboxylic acids successfully reacted with arylsulfonyl-substituted furoxans to give the corresponding alkyl-substituted furoxans with moderate to good yields. Various functional groups such as ketone, ether, ester, bromo, amine, and aromatic rings, as well as several bioactive and drug skeletons, were well tolerated in this method. Notably, this protocol showed exclusive site-selectivity favoring the cleavage of sulfonyl group at the 3-position of disulfonyl furoxan. The obtained 3-alkyl-4-sulfonyl furoxan products could be further converted into dual-functionalized furoxans, enabling the modular synthesis of diverse functionalities through “build-and-scrap” strategy.

**Scheme 15:**
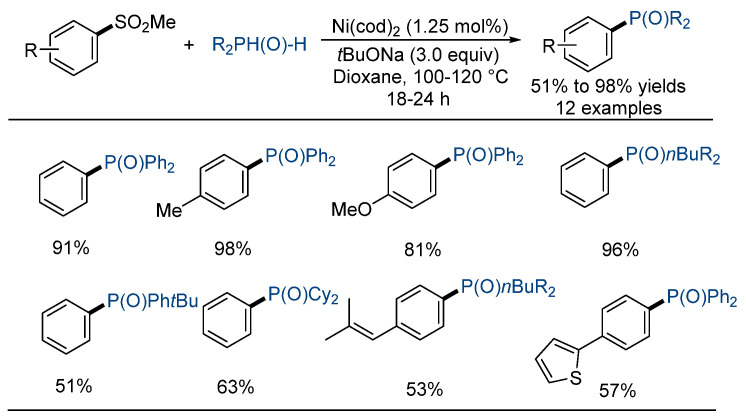
Ni-catalyzed intramolecular desulfonylative cross-coupling of *N*-hetero-2-aryl arylsulfones.

In recent years, the transition metal-catalyzed desulfonylative functionalization reactions to form carbon–heteroatom bonds have also been realized. In 2016, Chen and co-workers developed an efficient Ni-catalyzed desulfonylative cross-coupling of arylsulfones with phosphine oxides (Scheme 15) [30]. This protocol proceeded with both aryl and alkyl secondary phosphine oxides to offer functionalized *tertiary*-phosphine oxides in good to high yields. The desulfonylative reaction could be performed at 10 mmol scale in high yield with the addition of Ni(cod)_2_ at a loading of 0.1 mol%. Interestingly, the desulfonylative reaction of arylsulfones bearing ester and cyano groups could work well without nickel catalyst. This protocol provides an efficient method for the construction of C–P bonds. Later, the formation of C–B bond was achieved by the Yorimitsu group through Pd-catalyzed coupling of diphenyl sulfone with diboronates, yet with only one example [31]. 

**Scheme 16:**
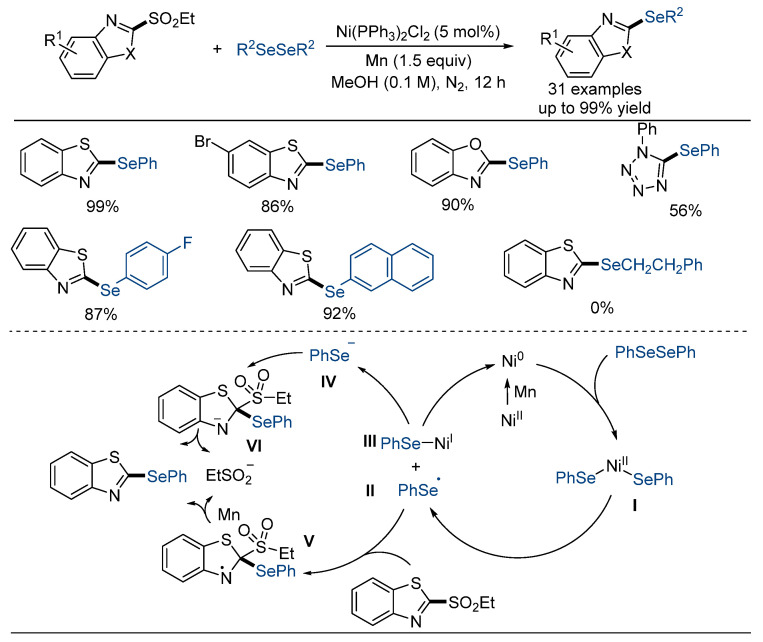
Ni-catalyzed desulfonylative cross-coupling with diselenides.

Organic selenium compounds have attracted a lot of attention due to their unique biological activity. Recently, the Wang group developed a Ni-catalyzed cross-coupling reaction of heteroaryl sulfones with diselenides (Scheme 16) [32]. Using 5 mol% of Ni(PPh_3_)Cl_2_ as catalyst, excess amounts of Mn powder as reductant, and MeOH or DMF as solvent, various heteroaryl sulfones and diselenides were well compatible to afford the anticipated heteroaryl selenides with moderate to high yields. In this protocol, the nickel catalyst undergoes oxidative addition with diselenide ether to furnish Ni(II)-selenide species **I**. Then, Ni(II)-selenide species can generate the selenium radical **II** and Ni(I)-selenide species **III**, which regenerates active Ni(0) species and the selenium negative ion **IV**. Finally, radical/nucleophilic addition into C=N double bonds within *N*-heteroarylsulfones followed by rearomatization releases the sulfite radical/negative ion and the target heteroaryl selenides.

## 3. Photo-/Electrocatalytic Desulfonylative Cross-Coupling

Over the past decades, photoredox catalysis has emerged as a powerful and promising strategy in organic synthesis [33,34]. Photocatalysis enables reactivity up-conversions by harnessing the energy of visible light and has been recently applied in a series of radical desulfonylative functionalization of sulfones. Recently, photoinduced desulfonylative functionalization that involves the cleavage of (hetero)aryl C(*sp^2^*)–SO_2_ bond of sulfones has also been achieved, providing a powerful and efficient route to access functional aromatics.

### 3.1. Desulfonylative Cross-Coupling with Alkylating Agents

**Scheme 17:**
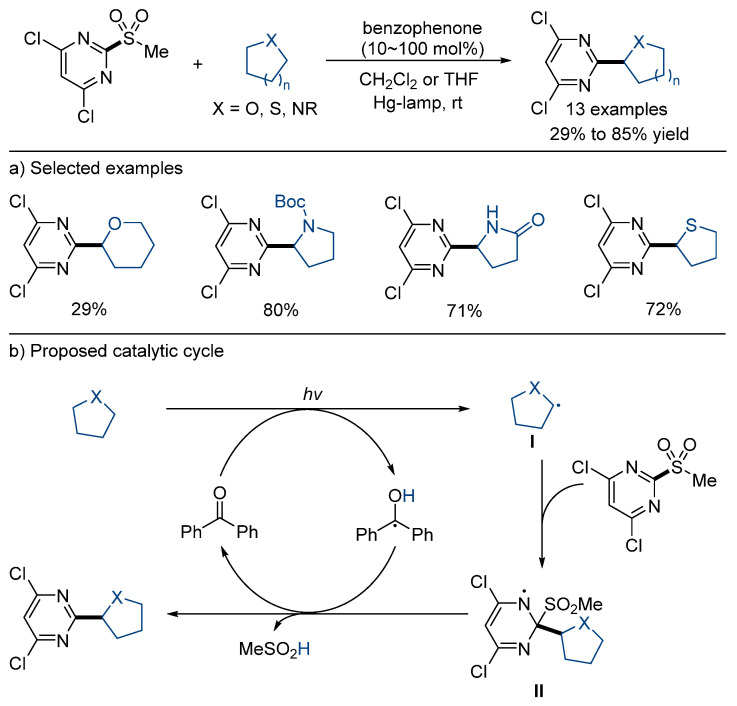
Ph_2_CO-mediated photoinduced desulfonylative alkylation.

In 2017, the Kamijo group reported a Ph_2_CO-mediated photoinduced desulfonylative alkylation of sulfonylpyrimidines (Scheme 17) [35]. In the presence of 0.1 or 1.0 equivalent of Ph_2_CO, the reactions could give various alkylated pyrimidines with moderate to good yields under the irradiation of medium-pressure Hg-lamp at room temperature. In this case, only electron-deficient substrates showed relatively higher yields. A proposed catalytic cycle was illustrated in Scheme 17b. Hydrogen atom abstraction (HAA) of the C(*sp^3^*)–H assisted by photoexcited diaryl ketone with the release of the ketyl radical occurs to generate a carbon radical **I**. The addition of the carbon radical to sulfonylpyrimidines furnishes the radical intermediate **II**, which undergoes rearomatization in the presence of ketyl radical to provide the desired product.

**Scheme 18:**
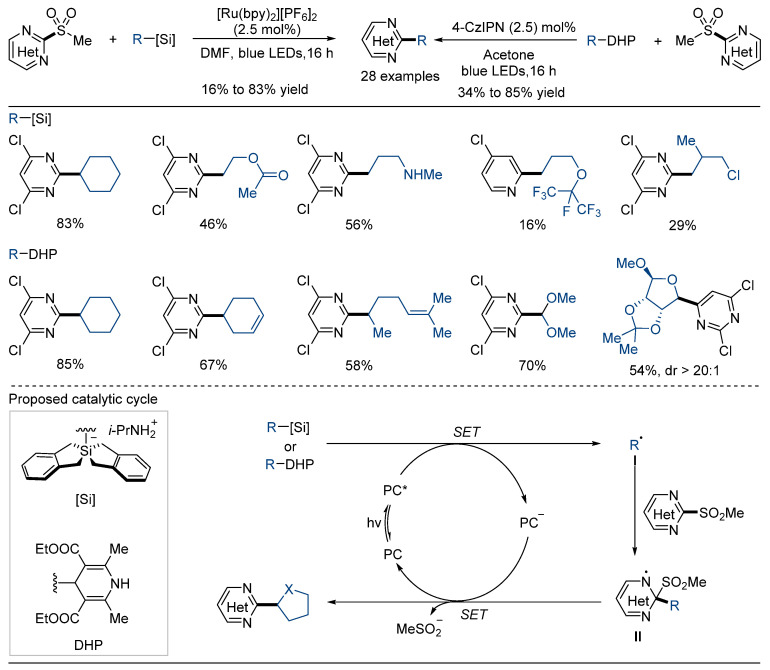
Photoinduced desulfonylative alkylation using functionalized reagents.

In 2019, Molander and co-workers reported a photoredox-catalyzed desulfonylative alkylation of *N*-heteroaryl sulfones with alkyl bis(catecholato)silicates or 4-alkyl-1,4-dihydropyridines (DHPs) (Scheme 18) [36]. When using alkyl bis(catecholato)silicates with low oxidation potentials as substrates, the desulfonylative reactions gave the best results in the presence of [Ru(bpy)_3_][PF_6_]_2_. Meanwhile, a variety of 4-alkyl-1,4-dihydropyridines also successfully reacted with the *N*-heteroaryl sulfones to yield comparable yields using 4CzIPN as the photocatalyst. Primary and secondary alkyl substrates bearing potentially reactive functional groups such as alkenes, esters, alkyl chlorides, amines, and pyrroles were well tolerated. To check the utility of this method, the desulfonylative reaction of a saccharide-derived DHP was subjected to the optimized conditions, yielding the final product in 54% yield with excellent diastereoselectivity. In the proposed cycle, excited photocatalyst by light absorption, followed by SET to the alkyl radical precursor. The newly formed alkyl radical inserts into the heteroaryl sulfone at the α-position relative to the sulfone with the concomitant elimination of a sulfinate anion by SET between the transient aryl radical and the reduced photocatalyst.

**Scheme 19:**
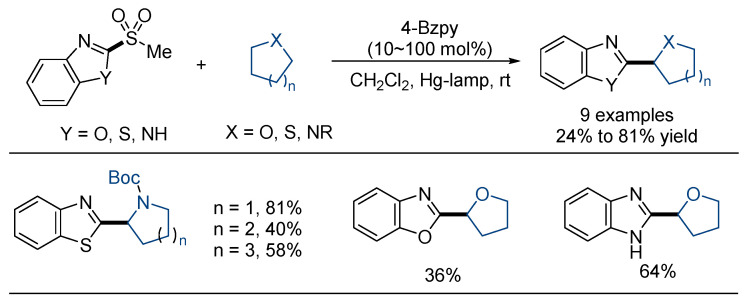
4-BzPy-promoted photoinduced desulfonylative alkylation.

In 2019, the Kamijo group developed similar photoinduced desulfonylative alkylation of sulfone-functionalized benzazoles mediated by 4-benzoylpyridine (4-BzPy) (Scheme 19) [37]. Sulfone-functionalized benzothiazoles, benzoxazole, and benzimidazoles could react successfully to give the desired products in 24–92% yield. However, this protocol required stoichiometric amounts of 4-BzPy as the photocatalyst due to its decomposition during the reaction. Recently, the Xia and Yang group achieved similar transformation through a photoinduced Fe-catalyzed ligand-to-metal charge transfer strategy [38]. In this method, the generated chlorine radical served as the hydrogen atom absorbing reagent.

**Scheme 20:**
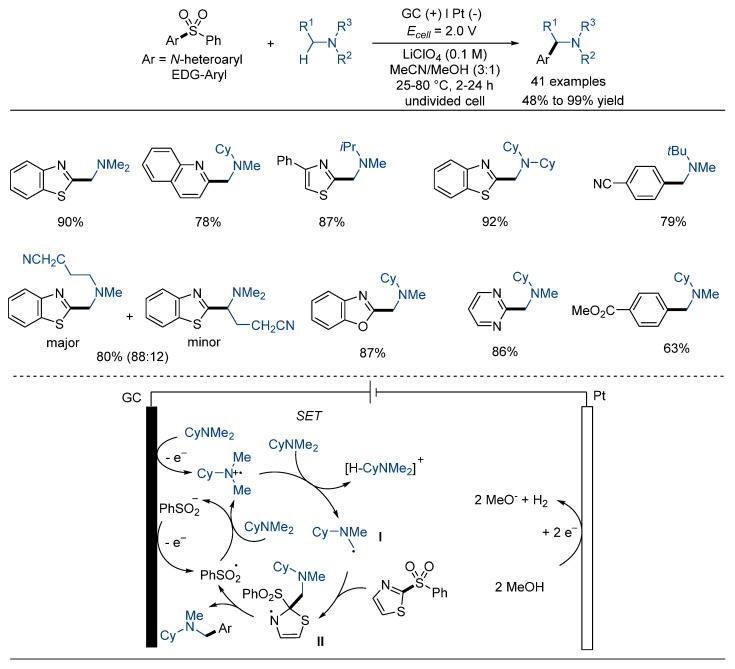
Electroinduced direct desulfonylative alkylation with amines.

In 2023, Shirakawa and co-workers developed an electrochemical direct *α*-arylation of trialkylamines with (hetero)arylsulfones (Scheme 20) [39]. By using a glassy carbon (GC) anode and a platinum (Pt) cathode in an undivided cell and lithium perchlorate (LiClO4) as a supporting electrolyte, the reactions successfully yielded model product in 97% yield under a constant cell voltage of 2.0 V (corresponding to an anodic potential of +0.86 V vs Ag/AgNO_3_) in a MeCN/MeOH (3:1) solution at 25 °C for 2 h. This protocol was compatible with diverse alkylamines and (hetero)arylsulfones containing various functional groups and aromatic rings. When using aryl(dimethyl)amines as substrates, one equivalent of 2,2,6,6-tetramethylpiperidine (TMP) was employed as the base to obtain a higher yield. The reaction is initiated by the direct oxidation of amines on a glassy carbon (GC) anode to give a cation radical followed by deprotonation process to form α-aminoalkyl radical **I**. Generally, a sterically compact carbon radical is kinetically favored to form. Then, the reaction of intermediate **I** with sulfones through homolytic aromatic substitution (HAS) yields the final product with the release of PhSO_2_^•^, which reacts with amines to form PhSO_2_^−^ and a cation radical. Very recently, a similar aminomethylation of sulfonylthiazoles enabled by the photoredox strategy has been achieved by the Fan and Chen group [40].

### 3.2. Desulfonylative Cross-Coupling with Alkenes

**Scheme 21:**
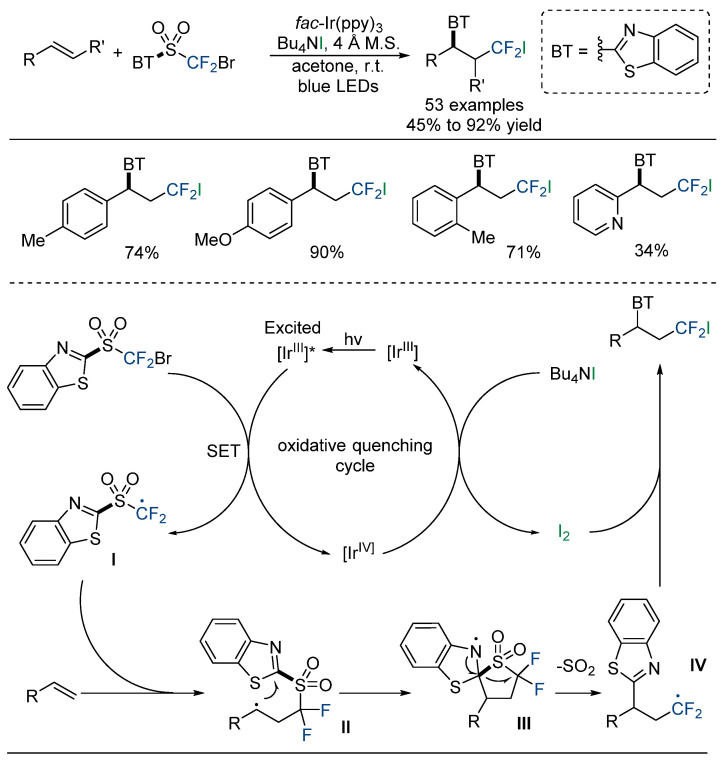
Photocatalytic desulfonylative dual functionalization of alkenes.

Alkenes are ubiquitous in natural products and pharmaceuticals and represent useful feedstocks in synthetic chemistry. The dual difunctionalization of alkenes provides a powerful route for olefin utilization. In 2018, Zhu and co-workers developed a conceptually new docking–migration strategy for radical difunctionalization of alkenes using bromodifluoromethyl *N*-heteroaryl sulfones (Scheme 21) [41]. This protocol employed *fac*-Ir(ppy)_3_ as photocatalyst (2 mol%) and blue LEDs as light source (14 W). Under the standard conditions, both aryl alkenes and inactivated aliphatic alkenes were suitable substrates for this reaction, leading to formation of diverse heteroaryl- and difluoromethyl-difunctionalized alkanes. It is noteworthy that the final products could be further converted into synthetically valuable fluorine-containing molecules. As shown in Scheme 21, the bromodifluoromethyl *N*-heteroaryl sulfone undergoes SET process with excited Ir(III)* to generate difluoromethyl radical species **I** and Ir(IV) complex. Then, addition of **I** to an alkene gives the active alkyl radical intermediate **II**, which simultaneously undergoes intramolecular cyclization to form a five-membered spiro-*N*-radical **III**. Next, aromatization and extrusion of SO_2_ results in the difluoroalkyl radical **IV**. Finally, the in situ formed iodine from the reaction of TBAI with Ir(IV) complex reacts with intermediate **IV** to yield the final difunctionalized product and regenerate the Ir(III) catalyst.

Later, the same group utilized easily accessed, bromoalkyl *N*-heteroarylsulfones as the dual-functional reagent, and developed a new “polarity umpolung” strategy for radical alkylation/*N*-heteroarylation of alkenes [42]. The *tert*-dodecylthiol was crucial for this transformation reaction as it served as both the hydrogen source and the reductant. Notably, conjugated dienes and enynes were well compatible with the protocol with high regioselectivity. Importantly, radical trapping agents such as PhSO_2_SPh, PhSO_2_SePh, PhSO_2_SCF_3_, CH_2_Br_2_, allyl sulfone, and even nitrogen-containing heteroarene, could also give dual functionalized products in satisfactory yields.

**Scheme 22:**
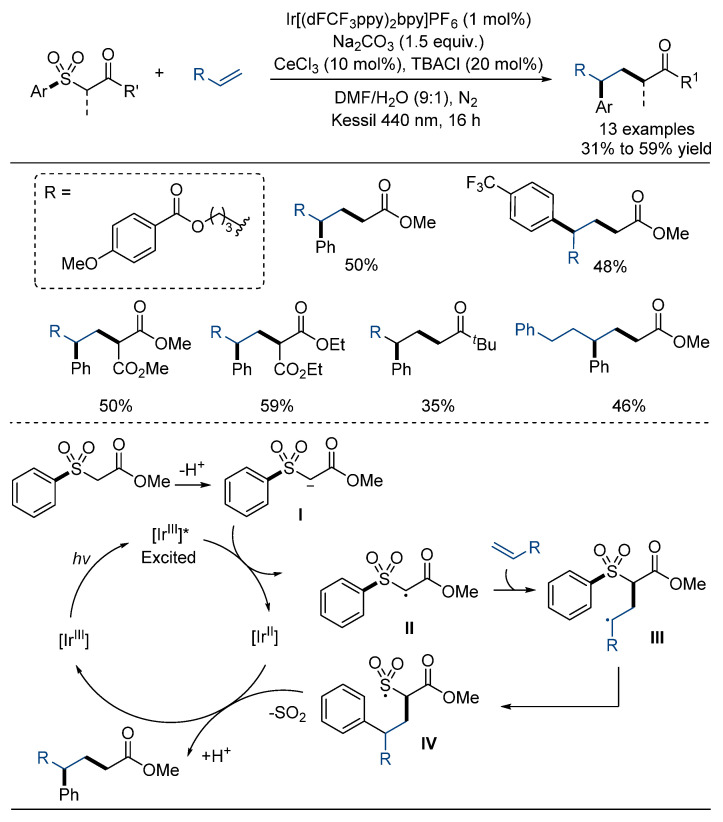
Photocatalytic desulfonylative dual functionalization of alkenes.

Very recently, Hong and co-workers reported a photocatalytic desulfonylative carboarylation of alkenes using arylsulfonyl acetates as both arylating and carbonylating reagents (Scheme 22) [43]. Both activated and nonactivated alkenes are proved to be suitable substrates to access the corresponding aryl esters. This reaction is proposed to initiate with a single-electron oxidation between the excited Ir(III)* species and the deprotonated arylsulfonyl acetate **I**, yielding the carbonyl radical **II**. Then, addition of intermediate **II** to an alkene affords an intermediate **III**, which undergoes intramolecular radical addition at the *ipso*-position regioselectively followed by homolytic cleavage of the C–SO_2_ bond to give the intermediate **IV**. Finally, the SO_2_ extrusion and single-electron oxidation in the presence of Ir(II) yield the desired product through proton transfer. This method offers an effective route for rapidly increasing molecular complexity with two valuable groups.

## 4. Conclusion

In summary, we have demonstrated desulfonylative functionalization reactions of organosulfones through the selective cleavages of inert (hetero)aryl C(sp^2^)−SO_2_ bonds. Since the seminal achievement in the 1970s, various desulfonylative cross-coupling reactions have been developed to form valuable carbon–carbon and carbon–heteroatom bonds, along with the discoveries of novel catalytic protocols including transition metal catalysis, photocatalysis, and others. Mechanism studies showed the involvement of (hetero)aryl-metal-SO_2_ species or radical processes. Despite the excellent progress in this field, several aspects should be stressed in developing new desulfonylative functionalization reactions in the future. For example, the cleavage of aromatic C(*sp^2^*)−SO_2_ bonds in unsymmetrical diarylsulfones generally tended to occur at the one linked to more electron-withdrawing substituents. For the desulfonylative functionalization of aryl sulfones, specific directing groups or alkyl substituents or harsh conditions such as the use of excess amounts of organometallic reagents or reductants, high catalyst loadings, and high temperature are usually required. Due to the inert reactivity of (hetero)aryl C(sp^2^)−SO_2_ bonds, examples of desulfonylative cross-coupling reactions to afford carbon–heteroatom bonds are much limited. Therefore, expansions of the sulfone substrate and reaction scopes, employments of milder reaction conditions with the developments of innovative catalytic protocols will be crucial in the future.
